# HE4 in the Differential Diagnosis of a Pelvic Mass: A Case Report

**DOI:** 10.3390/ijms12010627

**Published:** 2011-01-18

**Authors:** Emanuela Anastasi, Teresa Granato, Anna Coppa, Lucia Manganaro, Giuseppe Giannini, Sara Comploj, Luigi Frati, Cecilia Midulla

**Affiliations:** 1 Department of Molecular Medicine, Sapienza University, Rome 00161, Italy; E-Mails: scomploj@yahoo.it (S.C.); luigi.frati@uniroma1.it (L.F.); cecilia.midulla@uniroma1.it (C.M.); 2 CNR IBPM, Consiglio Nazionale Ricerche, Rome 00161, Italy; E-Mail: teresa.granato@uniroma1.it; 3 Department of Experimental Medicine, Sapienza University, Rome 00161, Italy; E-Mails: anna.coppa@uniroma1.it (A.C.); giuseppe.giannini@uniroma1.it (G.G.); 4 Department of Radiological Sciences, Sapienza University, Rome 00161, Italy; E-Mail: lucia.manganaro@uniroma1.it

**Keywords:** HE4 tumor marker, high-risk patients, ovarian cancer, endometriosis

## Abstract

Neoplasms of the ovary present an increasing challenge to the physician. Neoplastic ovarian cysts can resemble endometriomas in ultrasound imaging and need to be carefully considered in the differential diagnosis. We report the case of a woman with a strong family history of hereditary breast and ovarian cancer, who presented with a pelvic mass. The young girl refused oncogenetic counseling and genetic testing, even though she had a 50% *a priori* probability of being a BRCA1 mutation carrier. Pelvic magnetic resonance imaging (MRI) and a comparative analysis of the serum concentration of HE-4 and CA125 biomarkers provided accuracy and sensitivity in the diagnosis of a benign ovarian pathology. Based on this experience, we propose that the sensitivity of a screening program based on a HE4 and CA125 assay and MRI in high risk patients with mutations in the BRCA1 and BRCA2 genes may be considered a useful pre-operative tool for the differential diagnosis of pelvic masses.

## 1. Introduction

Ovarian cancer (OC) is the second most common form of gynecological cancer and the first cause of death from gynecological malignancy in the western hemisphere. Epidemiologic studies have clearly established the role of family history as an important risk factor for OC and inherited, highly penetrant germline mutations of the BRCA1 and BRCA2 oncosuppressor genes are responsible for 5–10% of the OC cases [1,2].

OC is often diagnosed at an advanced stage due to the absence of early symptoms and to the inadequacy of available screening methods, resulting in a low survival rate. The median survival period ranges from 18 to 24 months with an 80% probability of disease recurrence within five years [3].

Validation of novel biomarkers might help improving the specificity and sensitivity of ovarian cancer diagnosis. In example, studies on the *Human epididymis specific protein* 4 (HE4) alone or in combination with CA125 are being extensively carried out [4–8].

Recently, we have shown that not only HE4 is expressed in the early stages of the disease, but it is also an early indicator of disease recurrence [9].

In this case report, we applied the determination of serum concentrations of HE4 and CA 125 to the diagnosis of a pelvic mass that appeared in a young patient at high-risk for ovarian cancer due to her family history of breast cancer associated to a pathogenic BRCA1 gene mutation.

## 2. Case Presentation and Discussion

**Figure 1:**
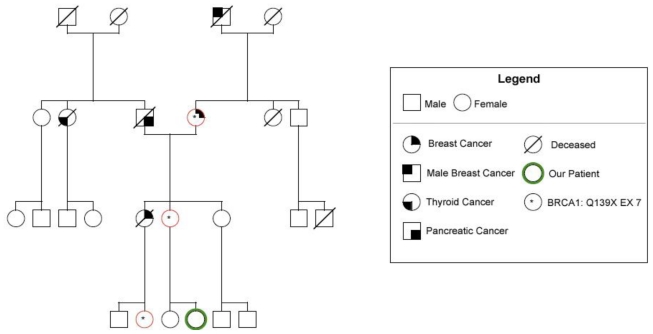
Genealogical diagram of the family with indication of the subjects mutated and the type of cancers.

A 24-year-old woman with a strong family history of breast cancer, and potentially at risk for breast and ovarian cancer due to a BRCA1 gene mutation (Q139X, Exon 7) discovered in her relatives (Figure 1), presented to our institution with a painful pelvic mass, with cyclic exacerbations during menses.

**Figure 2:**
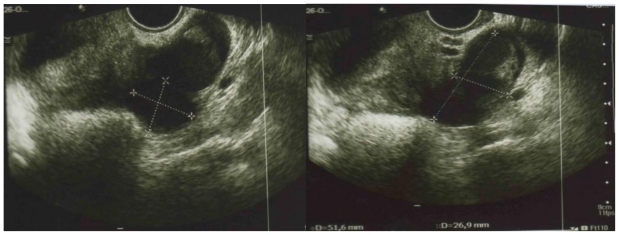
Transvaginal pelvic ultrasound shows cystic structures with low level internal echoes and echogenic wall foci, thickened walls and septations.

Transabdominal and transvaginal pelvic ultrasound, with the aid of a color Doppler imaging using a high-frequency probe (7.5 MHz), was performed for the diagnosis of a unilateral adnexal mass (Figure 2).

The ultrasonographic features showed cystic structures with low level internal echoes and echogenic wall foci, thickened walls and septations, and percystic color Doppler flow.

Despite the risk of being a mutation carrier due to the presence of the BRCA1 mutation in her mother (Figure 1), the young girl refused oncogenetic counseling and genetic testing. Informed of the possible diagnostic strategies, the girl also refused laparoscopic surgery.

**Figure 3:**
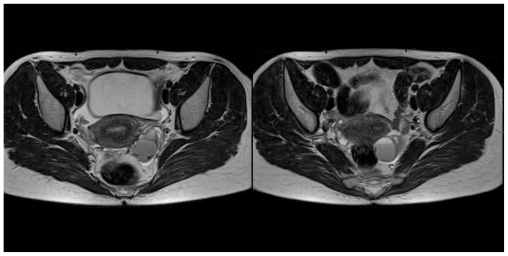
T2 TSE weighed sequences. Complex images in the left ovary, fluid/fluid level, hypointense images in the declive position, axially.

**Figure 4:**
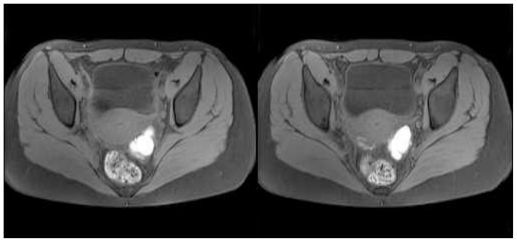
T1 VIBE weighed sequences. Marked hyperintense signal observed in the left ovary and a weakly hyperintense thin area is observed in the posterior uterine wall, referred to as an endometril implant.

Therefore, in order to improve diagnostic accuracy, we suggested pelvic magnetic resonance imaging (MRI) (Figures 3, 4) and determination of the serum concentrations of CA125 and HE4 biomarkers.

Single-shot and high-spatial-resolution with or without fat-suppressed T1 and T2 weighted sequences were performed with a 1.5T Siemens Avanto instrument, which showed several 3 cm cysts in the left ovary. They showed a hyperintense signal in the T2-weighted images for the presence of a fluid-fluid level and a strong hyperintense signal in the fat suppressed T1-weighted images, typical of endometriomas. Furthermore, similar signal characteristics were present in a thin area observed in the posterior uterine wall, referred to as an endometrial implant. In the lower part of the cysts, the hypointense T2-weighted images were referred to as blood clots.

Serum concentrations of CA125 and HE4 were analyzed by ELISA and, according to the manufacturer’s indications, the upper limits for normal values were considered to be 50 pmol/L and 35 U/mL for HE4, and CA125, respectively. Serum samples were collected both at the time of the gynecologic examination and two weeks later (Table 1). Coherent with the MRI finding, the low levels of HE4 tentatively indicated a non-malignant type of lesion.

This case of a young woman, potentially at genetic risk for breast and ovarian cancer because of her family history of breast cancer associated with a BRCA1 mutation, who presented with a pelvic mass of uncertain nature, exemplifies the diagnostic challenge that might occur in coping with subjects at genetic risk for this type of neoplasia.

BRCA1 mutation carriers experience a significantly higher probability of developing OC compared to the general population [2]. Although our patient had a 50% *a priori* probability of being a BRCA1 mutation carrier, due to presence of the familial mutation in her mother, she refused oncogenetic counseling and genetic testing, thus limiting our ability to more precisely define her individual risk. Therefore, urging the need to pose a correct diagnosis of the pelvic lesion, we approached the case by combining ultrasonography, MRI and determination of the serum levels of HE4 and CA125 biomarkers. The high and increasing levels of CA125 and the stably low levels of HE4 together with the features of the lesion from the MRI imaging suggested the occurrence of endometriosis. A laparoscopic study accepted by the patient at a later stage finally confirmed this evaluation.

A noninvasive means of discriminating between malignant pelvic masses and benign lesions is important given that approximately 20% of women will develop an ovarian cyst or pelvic mass at some point in their lives. Since serum based assays are noninvasive and relatively inexpensive, it would be ideal to have a biomarker or multimarker panel with sufficient sensitivity and specificity, which are able to discern the malignant potential that significantly improves differential diagnosis of pelvic masses.

Coupling these markers to techniques such as transvaginal sonography and the more expensive MRI increase the overall positive predictive value [10–12]. Based on this experience, we propose that determination of HE4 and CA125 serum concentrations together with pelvic ultrasonography and MRI may represent a diagnostic strategy for the early diagnosis of ovarian pathologies, in particular for individuals with a family history of breast/ovarian cancers associated with BRCA1/2 mutations, integrating previously defined intensified surveillance approaches [2]. Aware of the need to support such a proposal with prospective studies, we are planning to set a diagnostic trial to define the real cost/benefit of combining pelvic MRI and HE4 and CA125 serum determination in the standard screening of subject at high-risk for ovarian cancer, such as those with BRCA1 and BRCA2 mutations.

## 3. Conclusions

We conclude that the association of HE4 and CA125 serum concentration may be used to increase the accuracy in the differential diagnosis of pelvic masses in patients at high risk for ovarian neoplasias.

## Figures and Tables

**Table 1:** CA125 (expressed as U/mL) and HE4 (expressed as pmol/L) levels, evaluated at a two different times (the first at the moment of the gynecologic examination and the second two weeks later).

	Sample 1	Sample 2
**CA 125** (U/mL)	185	330
**HE4** (pmol/L)	44	40
